# The Use of Human Amniotic Membrane Tissue Grafting in Acute Traumatic Finger Injury: A Case Report

**DOI:** 10.7759/cureus.56177

**Published:** 2024-03-14

**Authors:** Jose V Perez, Mahi Basra, Krina Patel, Timothy Ganey, Lisa Finstein

**Affiliations:** 1 Wound Care, Curisec Wound Physicians, Miami, USA; 2 Osteopathic Medicine, Nova Southeastern University, Clearwater, USA; 3 Osteopathic Medicine, Nova Southeastern University, Miami, USA; 4 Clinical Management, Vivex Biologics, Miami, USA

**Keywords:** epithelization, acute trauma, vivex, cygnus matrix, placental tissue, acute traumatic wound, allograft tissue, human amniotic membrane, wound care management

## Abstract

Human amniotic membrane (hAM) is a collagen-based extracellular matrix that facilitates regenerative wound care. hAM offers several advantageous properties that promote epithelial cell growth, granulation, and angiogenesis. This case report demonstrates how Vivex Cygnus Matrix (Vivex Biologics, Miami, FL, USA) amniotic membrane was used over four weeks to graft a traumatic index finger injury that occurred while fishing. Cygnus Matrix allograft was first placed 72 hours after the accident. Following graft placement, the patient noted an immediate relief in pain and was able to return to all normal daily work activities within 48 hours of graft placement. Granulation tissue appeared a few days later. A total of four grafts were placed over the course of four weeks starting on September 4th, 2023. Typically, acute traumatic wounds are managed with a regimen of irrigation, wound dressing, and debridement. In this unique case, a distal fingertip amputation was treated with Cygnus Matrix allograft. A single hAM was applied weekly over the course of four weeks. Complete reepithelization of the injury was achieved with minimal scar formation. This paper demonstrates the use of hAM in healing acute traumatic wounds as an effective alternative to other more traditional treatments such as skin grafting, surgical reimplantation, and composite grafting. Utilization of hAM in acute traumatic wounds has few research reports that assure that the applications have minimal drawbacks while at the same time promoting wound management and patient comfort.

## Introduction

Human amniotic membrane (hAM) is a collagen-based extracellular matrix derived from human placenta. As a natural tissue product, hAM has contributed to tissue reengineering with a biologic impetus to support regenerative wound care [1]. With an inherent composition of cytokines and growth factors, placental membranes offer a robust basis for healing because of non-immunogenic properties. These properties afford a wide variety of clinical utilizations that can be incorporated into therapeutic care [2].

Human placenta consists of an outer amnion layer and inner chorion layer. The outermost tissue layer of the amnion consists of cuboidal epithelium firmly attached to a thick basement membrane and an underlying inner stroma and avascular connective tissue matrix [1]. The basement membrane of hAM supports adhesion, creates a barrier, and facilitates the migration of epithelial cells, supporting wound healing [3]. Human placenta has anti-inflammatory properties that can be attributed to a diverse composition of cytokines such as transforming growth factor-beta (TGF-B), fibroblast growth factor (FGF), granulocyte colony-stimulating factor (GCSF), interferon-gamma (IFNy), Interleukins-IL-1a, IL-1b, IL-4, IL-15, IL-16, and IL-17. Cytokines and growth factors such as basic fibroblast growth factor (bFGF), epidermal growth factor (EGF), fibroblast growth factor 4 (FGF-4), growth hormone (GH), insulin-like growth factor 1 (IGF-1), vascular endothelial growth factor (VEGF), and transforming growth factor alpha and beta (TGF-a, TGF-B) have been shown to promote tissue healing [1-4]. Lack of TGF-B activity has been implicated as a cause of chronic non-healing wounds [5]. Additionally, VEGF is an essential component of early angiogenesis in wound healing. FGF works in conjunction with VEGF to promote angiogenesis. FGF also allows for migration of essential cells like macrophages and fibroblasts and assists in re-epithelialization at wound site [5]. As a biologic tissue with inherent potential for aiding wound healing, hAM offers an alternative to local tissue transplant and skin grafting. hAM might provide an effective choice to physicians seeking therapeutic products to heal traumatic wounds.

Despite an extensive literature review, no references of hAM treatment as a biologic tissue replacement in traumatic finger wounds were found. Due to the aforementioned benefits of efficacious epithelization and anti-inflammatory properties, hAM provides an ideal traumatic wound healing modality. This case report presents a patient who suffered distal index fingertip amputation that was subsequently treated with Cygnus® Matrix allograft (Vivex Biomedical, Miami, FL, USA). Cygnus matrix allograft was chosen in this case due to availability and similarity of the product to other amniotic membrane allografts on the market. 

Cygnus Matrix meets United States Food and Drug Administration (FDA) approval under the criteria of minimal manipulation and homologous use as a biological membrane covering that provides an extracellular matrix scaffold. Cygnus Matrix supports the patient’s ability to repair underlying damaged tissue. As an amniotic tissue allograft, Cygnus Matrix retains the features of intact human amniotic membranes; specifically assuring that the amnion and chorion layers are never separated. In preserving the anatomy, multiple extracellular matrix proteins, growth factors, collagen, cytokines, and other specialty proteins that are intrinsic to wound repair are maintained. The amniotic tissues are supplied through a donation process and undergo processing that removes blood remnants while preserving the allograft composition. This allows for maintenance of key extracellular matrices including proteins, carbohydrates, growth factors, and cytokines that support regenerative potential in therapeutic use.

## Case presentation

**Figure 1:**
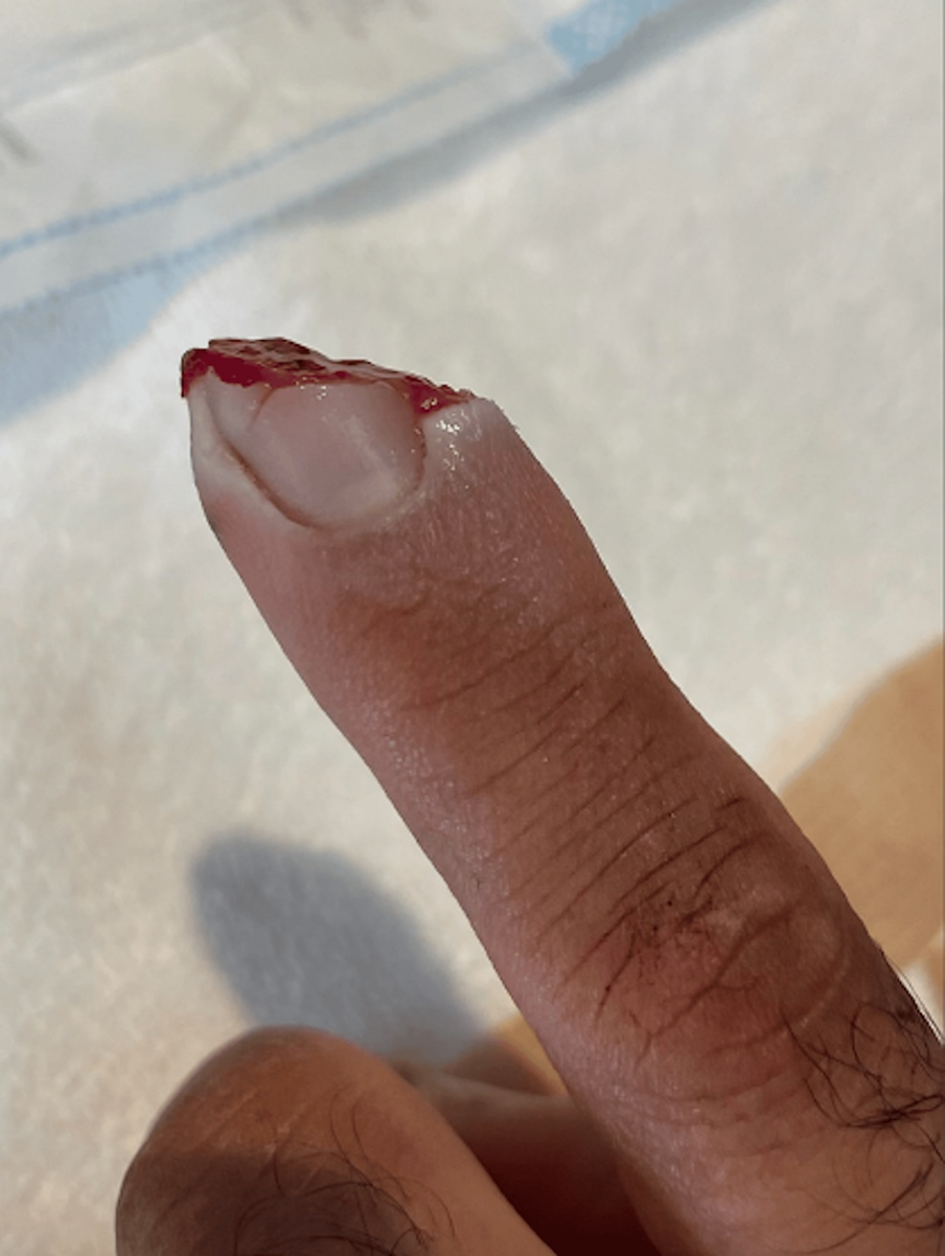
Left index finger after traumatic injury

**Figure 2:**
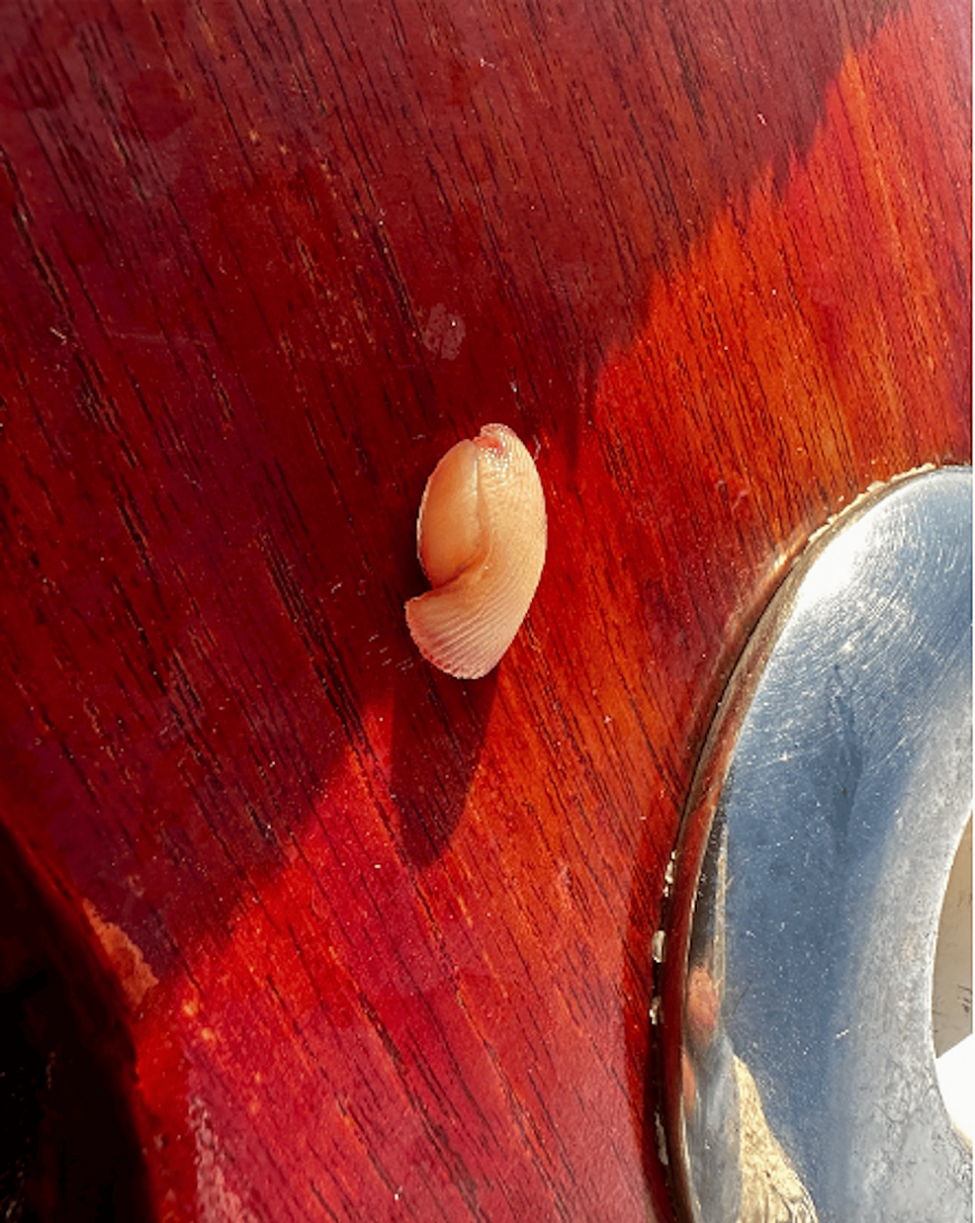
Traumatic amputation of left index finger

A 52-year-old male presented to the office of Dr. Jose Perez following a traumatic injury to his left index finger while fishing. The distal part of his finger was sliced from being caught in fishing line. The patient's past medical history included hypercholesterolemia for 20 years with atorvastatin management. The patient uses alcohol occasionally and denies tobacco and illicit drug use. Upon examination, 2 cm of tissue of the distal left index finger was sliced at a 45-degree angle from a fishing line, as shown in Figure 1. The patient reports that immediate throbbing pain was felt after the injury on September 1st, 2023. 2 cm of his distal phalanx was on the floor of the boat, as noted in Figure 2. A tourniquet was placed proximal to the wound site. For pain management, he took Tylenol and Advil along with prophylactic antibiotics. A five-day course of Augmentin and Bactrim was completed.

Cygnus Matrix allograft was placed 72 hours after the accident by wound care specialist, Dr. Jose Perez. Following graft placement, the patient noted an immediate relief in pain. Granulation tissue appeared 28 days later. A total of four grafts were placed between September 4th, 2023, and September 22nd, 2023 with weekly placement. The patient was able to return to all normal daily work activities within 48 hours of graft placement. Other symptoms that were noted included hyperparesthesias in the entirety of his phalanx with light touch and some residual soreness.

**Figure 3:**
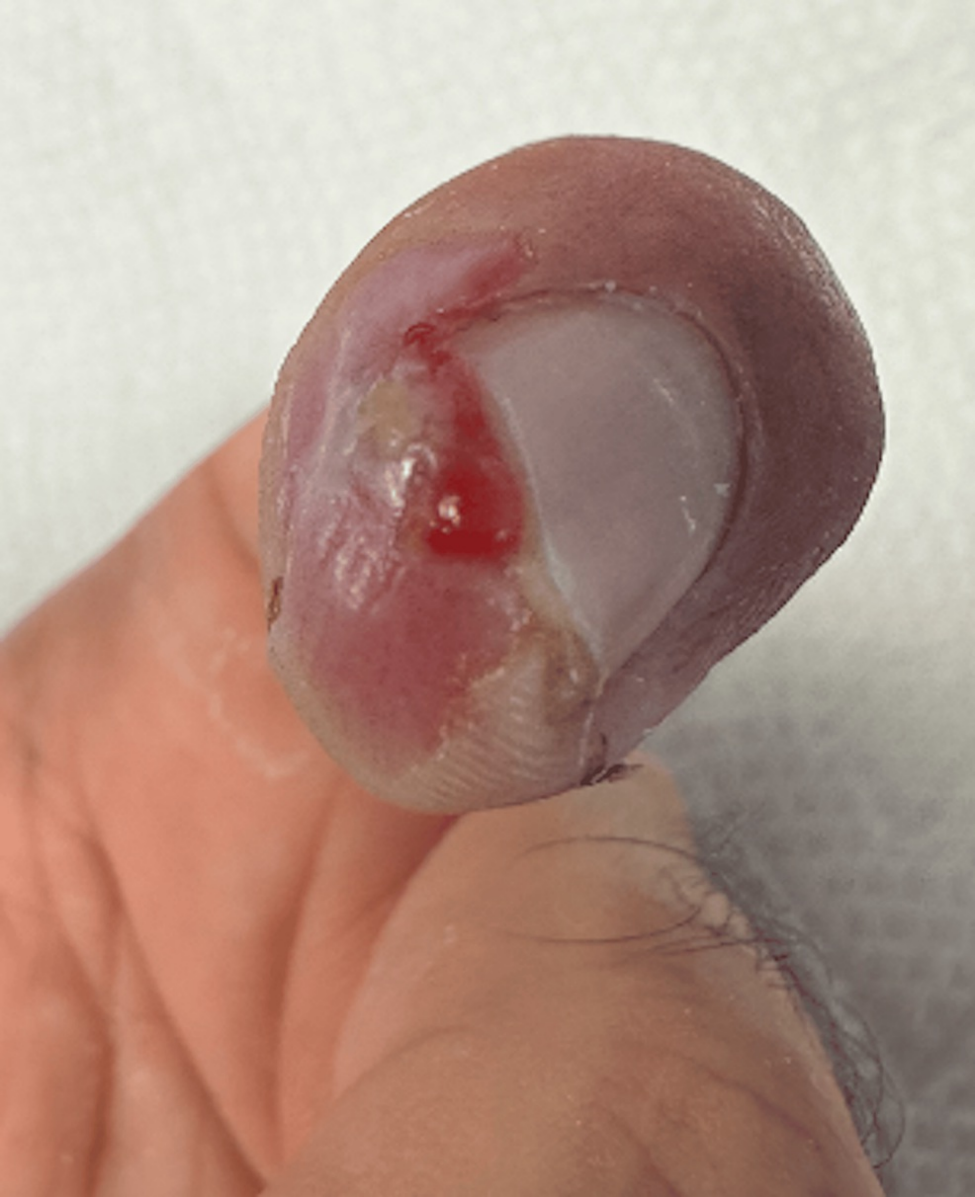
On October 6th, a small granuloma formed at the tip of the fingertip can be seen. This was cauterized by silver nitrate.

**Figure 4:**
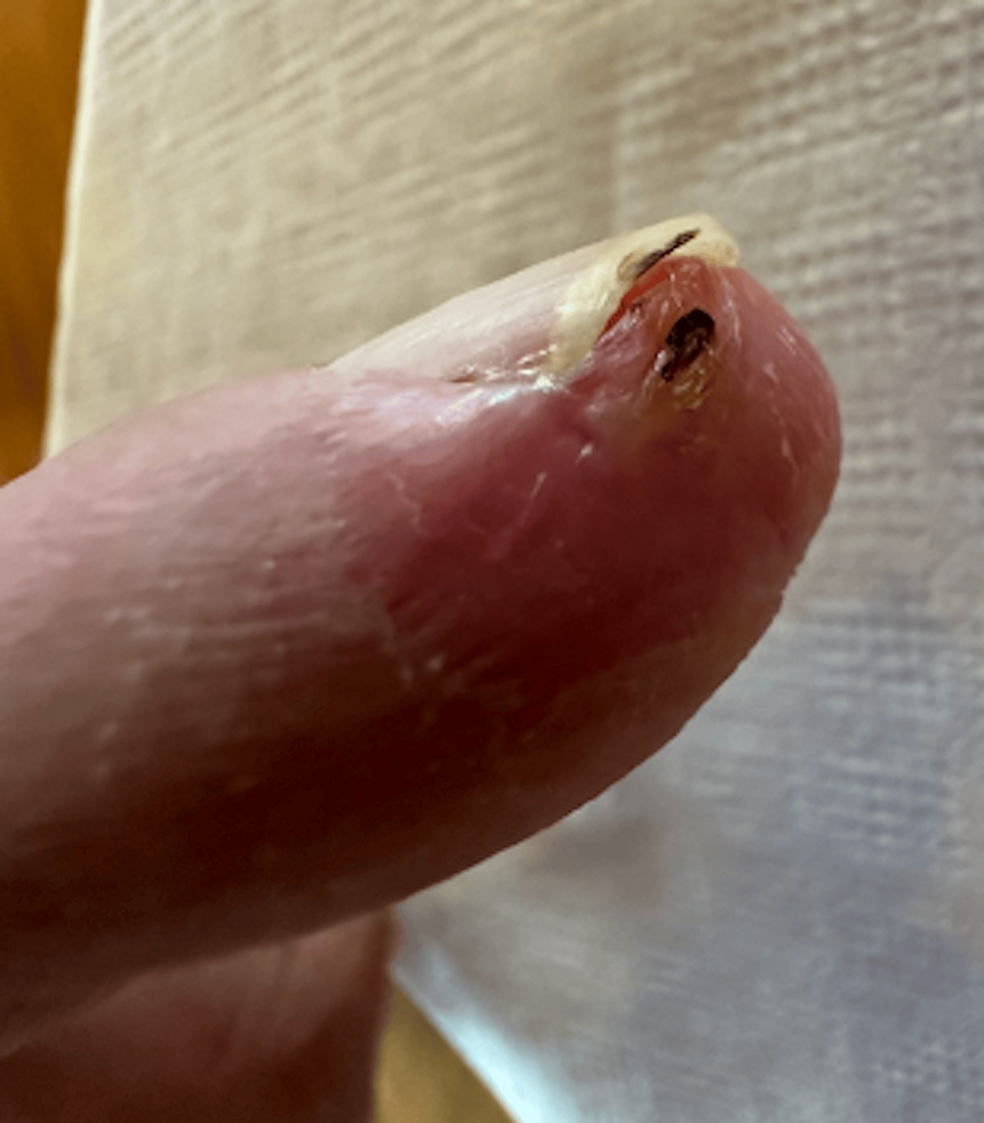
Resolution of wound on October 13th

**Figure 5:**
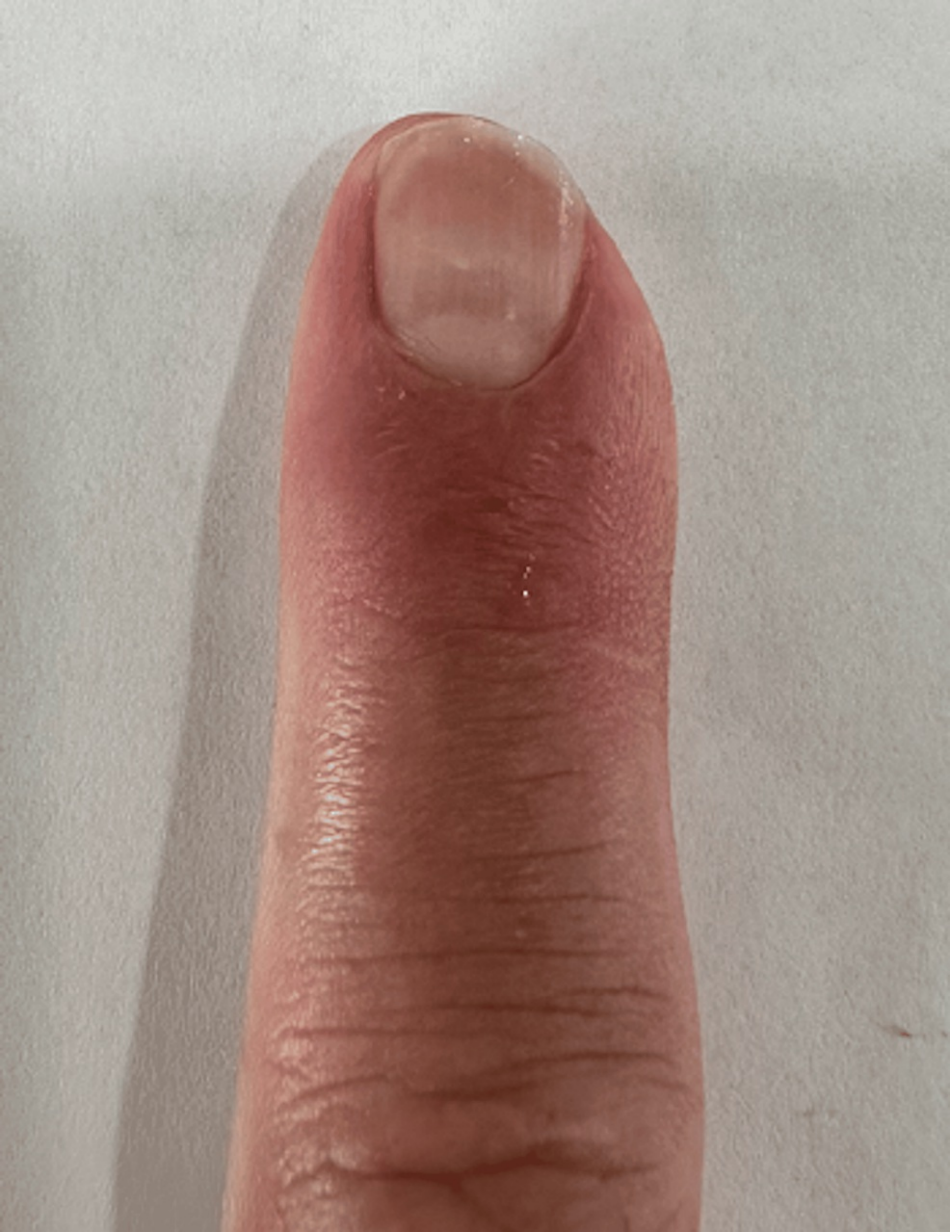
Posterior left index finger 46 days after injury

**Figure 6:**
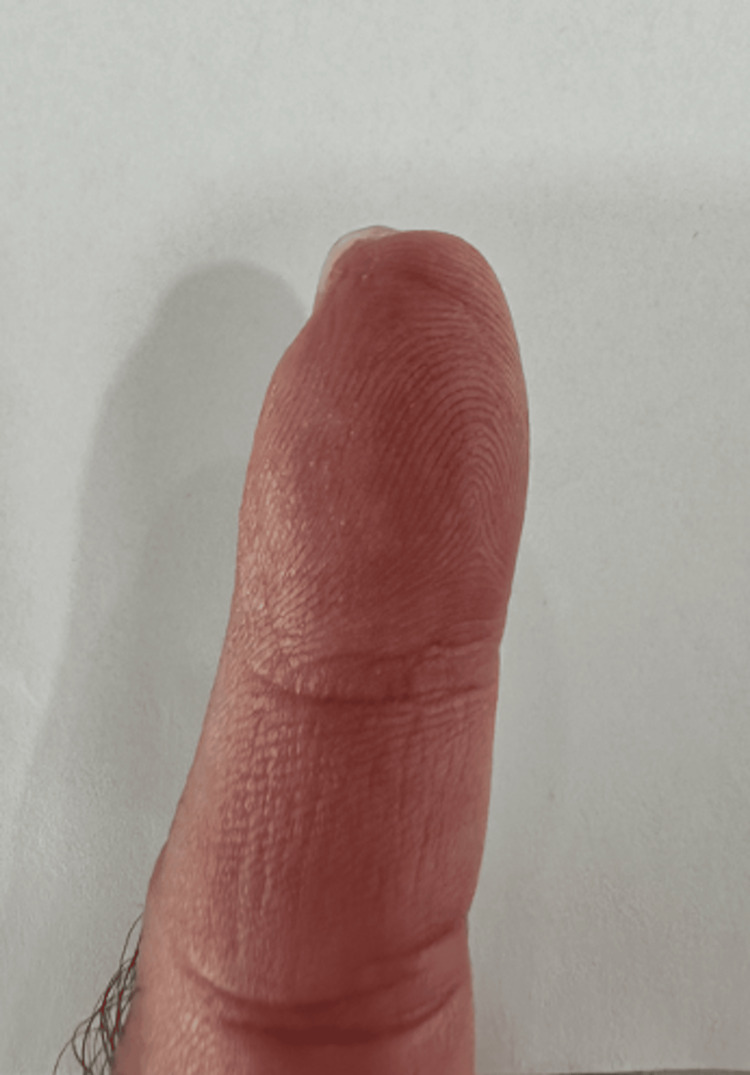
Anterior left index finger 46 days after injury

The remainder of the wound course was uneventful. Figure 3 displays the wound after 42 days once four grafts were placed. A small granuloma formed at the lateral side of the index finger which was cauterized with silver nitrate. Figure 4 shows the resolution of the wound, 49 days after application of graft. Figures 5-6 depict resolution of the wound. Consent was obtained from the subject for drafting and publishing this case report.

## Discussion

This case report highlights the potential of hAM in management of wounds that emerge from acute trauma. Previous research has shown a proven benefit in the management of chronic wounds using hAM that results in wound area reduction [6]. hAM improves outcomes in chronic non-healing wounds by promoting granulation, angiogenesis, and epithelization [7].

Typically, acute traumatic wounds are managed with a regimen of irrigation, wound dressing, and debridement [8]. In the case of a fingertip amputation, a typical plan of care would involve surgical replantation, composite grafting, or healing by secondary intention [9]. In this unique case, a distal fingertip amputation was treated with Cygnus Matrix allograft. A single hAM was applied weekly over the course of four weeks. Gradual decrease in wound area was noted. The application of hAM resulted in regeneration of the distal fingertip with epithelization and restoration of fingertip contour. The wound was completely resolved within five weeks.

Use of hAM may have additional benefits that aid in recovery for the patient by reducing pain sensation. During the course of treatment, this patient reported a minimal amount of pain and discomfort. The analgesic effects of placental tissue have been investigated in prior research and shown to be effective in reducing nociceptive signals. One such study proposed that lipid mediators such as palmitoylethanolamide (PEA), a lipid found in amniotic membrane, downregulates nociceptive signaling via activation of peroxisome proliferator-activated receptor alpha (PPAR- α) [10]. PPAR-α is a ligand activated transcriptional factor which modulates pain signaling [10]. Additional factors such as increased wound hydration, barrier protection, and decreased inflammation after application of hAM may also play a role in the reported attenuation of pain perception [6]. Additionally, N-acylethanolamides (NAEs) such as PEA, anandamide (AEA) and oleoylethanolamide (OEA) may play a role in the analgesic effects of hAM [11]. These NAEs are bioactive lipid mediators that play a role in immune function, pain and inflammation [11]. AEA alleviates pain by interacting with cannabinoid-1 receptors in the periphery as a powerful antinociceptive [12]. PEA is a selective agonist on peripheral cannabinoid 2-like receptors, inhibiting mechanical hyperalgesia [12]. 

Some limitations in the use of hAM include the possibility of infection from graft tissue and the lack of tensile strength [2]. There is a possibility of transmission of certain viruses from tissue grafting such as hepatitis and HIV [2]. However to date, there have been no reported disease transmissions from the application of Vivex Cygnus Matrix amniotic membrane allograft [13]. hAM is largely nonimmunogenic, thus there is limited chance of mounting an immune rejection to the graft [14]. This phenomenon may be attributed to the upregulated expression of human leukocyte antigen G (HLA-G) on amniotic membranes. HLA-G is responsible for suppressing macrophage and natural killer cell activity, contributing to low immunogenicity [14]. The fragility and limited tensile strength of the graft is a limitation that can be overcome if proper wound dressing is done to secure the site of application. Patient education is also important to avoid excessive friction and movement of the area. 

This case provides a look into the unexplored benefits of hAM in atypical cases. Despite the few limitations listed above there is an exciting future to be researched regarding the use of hAM in the treatment of acute traumatic wounds. Further research is required to better understand potential benefits of this therapy.

## Conclusions

Human amniotic membrane is a versatile tool that has traditionally been used in the management of chronic wounds but has unexploited benefits in the treatment of acute traumatic wounds. hAM assists in the formation of granulation tissue, angiogenesis and stimulates epithelialization. These factors may lead to a reduction of wound area with marked improvement of finger contour. Additionally, hAM likely provides a noticeable decrease in pain sensation and quick return of function as is the case with this patient. Utilization of hAM in acute traumatic wounds is an under-researched modality with minimal drawbacks with great potential to offer in terms of wound management and patient comfort.
